# Impact of the Coal Mining on the Spatial Distribution of Potentially Toxic Metals in Farmland Tillage Soil

**DOI:** 10.1038/s41598-018-33132-4

**Published:** 2018-10-08

**Authors:** Fang Li, Xinju Li, Le Hou, Anran Shao

**Affiliations:** 1 College of resources and environment, Shandong Agricultural University, Tai’an, 271018 China; 2 College of economics and management, Shandong Agricultural University, Tai’an, 271018 China

## Abstract

Coal mining areas are prone to hazardous element contamination because of mining activities and the resulting wastes, mainly including Cr, Ni, Cu, Zn, Cd and Pb. This study collected 103 samples of farmland tillage soil surrounding a coal mine in southwestern Shandong province and monitored the heavy metal concentrations of each sample by inductively coupled plasma mass spectrometer (ICP-MS). Statistics, geostatistics, and geographical information systems (GIS) were used to determine the spatial pattern of the potentially toxic metals above in the coal mining area. The results show that the toxic metal concentrations have wide ranges, but the average values for Cr, Ni, Cu, Zn, Cd and Pb are 72.16, 29.53, 23.07, 66.30, 0.14 and 23.71 mg Kg^−1^, which mostly exceed the natural soil background contents of Shandong Province. The element pairs Ni-Cu, Ni-Zn, and Cu-Zn have relatively high correlation coefficients (0.805, 0.505, 0.613, respectively). The Kriging interpolation results show that the contents of soil toxic metals are influenced by coal mining activities. Moreover, micro-domain variation analysis revealed the toxic metals in the typical area of the coal transportation line. These findings offer systematic insight into the influence of coal mining activities on toxic metals in farmland tillage soil.

## Introduction

As a global environmental issue, soil contamination has been increasingly recognized as a problem^1^ owing to the importance of soils for agricultural production as well as the maintenance of the health of plants, animals and human beings^2,3^. Nationally, soil is used at an overshoot rate of 16.1%. Higher overshoot rates can be observed in mining areas, and in China’s 2014 Soil Pollution Condition Investigation Communique, which was released by the Ministry of Land and Resources combined with Ministry of Environmental Protection, a typical plot reached an overshoot rate of 33.4%^4^. Soil pollution is one of the main environmental problems in the European Union. It is estimated that more than 2.5 million sites are potentially polluted, 60% of which are likely affected by hydrocarbons or trace elements^5^. Heavy metals generally refer to metals and metalloids that have densities greater than 5 g/cm^3^, such as lead (Pb), zinc (Zn), cadmium (Cd), mercury (Hg), chromium (Cr), arsenic (As) and so on^6^. Enrichment of heavy metals in soil may cause carcinogenic and mutagenic effects, which pose severe threats to the health of animals and humans exposed to the soil environment^7,8^.

Coal mining and consumption play a significant role in the economic and social development of China. Long-term intensive mining activity has severely disturbed the natural environment via subsidence, soil erosion, pollution and the deterioration of the water quality^9^. It is well known that various toxic elements exist in coal refuse and fly ash, and these toxic elements can be released and enter the soil through coal industry activities, including production and accumulation of a large amount of gangue, discharge of sewage, and emissions from coal-fired power plants and coal transportation^10^, all of which are a challenge to agricultural security as a result^11^.

From a recent literature review, it was shown that numerous studies of soil heavy metal pollution caused by mining activities have been carried out over the past few years. For instance, Ge *et al*.^12^ analyzed and assessed the ecological risk from seven heavy metals, namely, Cd, Hg, As, Cu, Pb, Cr and Zn, that were present in soils surrounding a coal waste pile at Naluo Coal Mine, Liupanshui, Guizhou, China. The results revealed that the heavy metals mentioned above were strongly elevated, if which Cd had the maximum single pollution index^12^. In a separate report, Niu *et al*.^13^ analyzed the heavy metals (Cu, Zn, Ni, Pb, Cr, Cd, and Hg) found in 33 surface soil samples from coal-mining land restored for use as cultivated land in Xinzhuangzi, China. The results showed that the selected elements were elevated, especially Cd^13^. In addition, Chen *et al*.^9^ collected 90 soil samples from different depths (0–20, 20–40, 40–60 cm) and 120 plant samples based on a grid sampling method in the coal-refuse reclaimed areas of Huainan, China, to investigate the concentration and distribution characteristics of toxic elements in soils and plants. The results showed that the concentrations of toxic elements (As, Cd, Cu, Ni, Pb and Zn) in the soils were elevated following coal refuse reclamation. Toxic element tolerance was observed in all of the selected plant samples, and the concentrations of toxic elements in underground tissues were higher than those of above ground tissues, with the concentrations varying from highest to lowest at depths of 20–40 cm, 40–60 cm, 0–20 cm^9^. Numerous studies of the enrichment content of heavy metals in crops related to mining activities have also been carried out. For instance, Tao *et al*. (2017) reported that the heavy metals concentrations (Pb, Cd and Cr) in mature rice in a coal mining area in Guizhou were 2~8 times higher than those of national health food safety standards limits^14^. In a separate report, Cheng *et al*. (2016) suggested that the contents of Cu, Cd and Pb exceeded the standard limits in some vegetables. The total risk from exposure to the multiple metals in vegetables exceeded the acceptable levels for both adults and children^15^. Moreover, a few studies on the enrichment content of heavy metals have focused on coal mine waste water^16^ or on dust surrounding a coal-fired power plant^17^. The studies mentioned above and many unmentioned studies show that attempts have been made to gain a broader understanding of the effect of coal activities on soil toxic metals, mostly by applying geostatistical methods. However, few studies have investigated how these activities interact with each other, as the distribution of soil toxic metals result from the combined impact of the above activities. Knowledge of the spatial distribution of toxic elements in soil in the entire mining area is necessary. Furthermore, the influence of coal transportation on heavy metals distribution has been ignored. Further research is needed in this regard.

Knowledge of the spatial distribution of toxic elements in soil, especially in farmland tillage soil, is necessary to assess the environmental hazard and strategy for dealing with this hazard. The concentrations of toxic metals must be monitored and assessed to determine the level of soil toxic metal pollution and prepare for remediation. Sampling analyses have to be performed to evaluate the concentrations of toxic elements due to geogenic (natural, background) or anthropogenic phenomena in special areas, such as a coal mining area^18,19^. However, soils have spatially variable characteristics, for which standard statistical methods are not applicable, and therefore, a set of statistical tools need to be used to describe these characteristics. A model of spatial dependence can be used to comprehensively determine the complex relationships between soil properties. These methods are accurate and well documented by researchers^20^. A few studies have been conducted to evaluate toxic element pollution and to explore the toxic element spatial distribution characteristics of coal mining areas in China over the past few years (2013–2017). The results show that in different coalfields, the amount of pollution from different heavy metals is quite different. The heavy metals most affected by coal industrial activities are Cd, Hg, Mn, Cu, Zn, Ni and Cr^21–24^. Thus, a comprehensive heavy metals pollution assessment of the Yanzhou coalfield is urgently needed.

Ren *et al*. believe that there are 22 types of harmful toxic elements in coal^25^. As is known, Hg has become the primary toxic element of concern in coal in China, as it is present at higher average concentrations than in the rest of the world. Much research has been performed to describe the release and distribution characteristics of Hg^26–28^. Thus, this paper investigates toxic elements (Cr, Ni, Cu, Zn, Cd, and Pb) in farmland tillage soil samples, which has been studied in relevant references of this area^29–32^. The main objectives of this study are 1) to investigate and determine the toxic element concentrations and evaluate the soil heavy metal contamination levels of a mine in Yanzhou coalfield; 2) to explore the spatial variation and distribution characteristics by applying a geostatistical method; and 3) to reveal the micro-domain variation of heavy metals in the periphery of a coal transportation road. It is expected that these findings will be used as management tools and environmental remediation strategies at coal mine sites.

## Results and Discussion

### Concentrations of toxic metals in soils

A statistical description of the elements in the research area is presented in Table 1. The background value of Shandong soil was used as the reference value. The arithmetic average concentrations of Cr, Ni, Cu, Zn, Cd and Pb were 72.16, 29.53, 23.07, 66.30, 0.14 and 23.71 mg/Kg, respectively. Compared to the background value, the soil in coal mine area had elevated concentrations of Cd, Cr, Ni and Zn. The maximum concentrations of Ni, Zn, Cd, and Pb were 62.08, 124.85, 0.7 and 57.34 mg/Kg, which were 2.7, 2.0, 8.3 and 2.2 times greater than the background values, indicating that these metals were derived from anthropogenic sources, particularly Cd, which had a mean concentration 1.7 times greater than the background value. We compared the toxic metal concentrations in soil from coal mine areas to those reported in previous studies. The mean concentrations of the six toxic metals in this study were slightly lower than those in Lianyuan^33^. The comparison between data in this study and other areas were shown in Table 2.

**Table 1:** Descriptive statistics and basic testing for soil toxic metals content.

Item	Cr	Ni	Cu	Zn	Cd	Pb
Number of sample point	103	103	103	103	103	103
Mean value (mg/Kg)	72.16	29.53	23.07	66.30	0.14	23.71
Maximum (mg/Kg)	97.05	62.08	39.48	124.85	0.7	57.34
Minimum (mg/Kg)	45.80	14.76	6.87	33.75	0.04	14.84
Media value (mg/Kg)	72.73	28.98	22.86	64.35	0.13	22.91
Background Value in Shandong (mg/Kg)	65.20	23.00	24.00	63.50	0.084	25.8
Average concentration in coal of this mine (mg/Kg)	68.28	134.68	121.93	684.11	0.40	225.75
Average concentration in coal gangue of this mine (mg/Kg)	88.40	40.4	48.65	768	2.50	65.5
SD	13.63	6.38	5.63	14.19	0.07	6.43
Skewness	−0.24	1.18	0.27	0.88	4.57	2.65
Kurtosis	−0.92	5.97	0.96	2.39	31.66	9.50
Coefficient of variation CV/%	19.44	19.09	24.71	22.39	30.77	23.24
Kolmogorov-Smirnov Z	0.729	0.886	0.98	0.774	1.645	2.113
Asymptotic significance (double side)	0.662	0.412	0.292	0.587	0.009	0

**Table 2:** Heavy metal concentrations (mg/kg) in coal mine area in China and other countries.

City	Toxic metals						Reference
	Cr	Ni	Cu	Zn	Cd	Pb	
Anhui(China)	—	—	36.8	62	—	25.4	^9^
Xinjiang (China)	48.83	24.18	36.97	62.48	1.09	—	^24^
Guizhou (China)	20.89	—	46.61	60.07	0.43	9.09	^12^
NeiMonggolAutonomousRegion (China)	—	27.32	17.06	56.74	0.06	12.21	^52^
Shanxi (China)	275	94.5	55	—	0.8	54.2	^37^
Yunnan (China)	148.27	110.59	191.05	2273.77	—	1117.47	^53^
Henan (China)	50.97	—	26.97	109.63	0.61	70.10	^54^
Beijing (China)	48.56	30.98	—	—	0.29	—	^55^
Rostoc Oblast (Russia)	200	40	20	50	—	10	^56^
Chhattisgarh (India)	567.4	—	218.3	426	—	311	^57^

To compare the variability of the soil toxic metals concentrations in the study area, the coefficient of variability (CV) was calculated and categorized into four classes according to previous studies^34,35^. CV ≤ 20% was regarded as low variability, 21% < CV ≤ 50% indicated moderate variability, 51% < CV ≤ 100% as regarded as high variability, and 100% < CV was considered very high variability. According to Table 1, the toxic metals of Cu, Zn, Cd and Pb showed moderate variability and CV values in the range of 21% to 50%. Cd had a CV of 30.77, which was the highest value among the toxic metals. The results further show that Cd-bearing soils in this research area are attributed to anthropogenic sources^36^. The CV values of Cr and Ni showed low variability, further indicating low anthropogenic import of these metals.

The skewness coefficient and kurtosis were used to describe the symmetry and shape of the toxic metal distributions. The skewness coefficients of Cr and Cu were near zero (the value of the standardized normal distribution of skewness is zero. Skewness >0 means the center is shifted to the left, while skewness <0 means the center is shifted to the right), while the other elements had high skewness, indicating disordered high values. The kurtosis of Cd was much greater than zero (the value of the standardized normal distribution of kurtosis is zero. Kurtosis >0 means the distribution has a towering shape, while Skewness <0 means the distribution has a flat shape). The hypotheses that the concentrations of Cd and Pb were subject to normal distributions were refuted because the asymptotic significance (double side) of the Cd and Pb distributions were both less than 0.1.

Correlation pairs for all elements were investigated, as shown in Table 3. Positively high correlation coefficient of variations for Ni and Cu were observed because their Pearson correlation was 0.805 (a significant correlation was found at the 0.05 level (bilateral)). Ni-Zn and Cu-Zn also had positive correlation coefficients. The results indicate that both elements have a similar behavior or arise from a similar source.

**Table 3:** Correlation table of toxic metal elements.

		Cr	Ni	Cu	Zn	Cd	Pb
Cr	Pearson correlation	1					
	Significance(double side)						
Ni	Pearson correlation	0.319^**^	1				
	Significance(double side)	0.001					
Cu	Pearson correlation	0.296^**^	0.805^**^	1			
	Significance(double side)	0.002	0.000				
Zn	Pearson correlation	−0.046	0.505^**^	0.613^**^	1		
	Significance(double side)	0.642	0.000	0.000			
Cd	Pearson correlation	0.048	0.208^*^	0.176	0.146	1	
	Significance(double side)	0.631	0.034	0.074	0.139		
Pd	Pearson correlation	−0.058	0.271^**^	0.290^**^	0.380^**^	0.064	1
	Significance(double side)	0.561	0.005	0.003	0.000	0.518	

**Significant correlation was found at the 0.01 level (bilateral). *Significant correlation was found at the 0.05 level (bilateral).

### Pollution assessment and geostatistical analysis

The geoaccumulation index of toxic metals is shown in Table 4. The *I*_*geo*_ average values for Cr, Ni, Cu, Zn and Pb in samples were generally less than 0, indicating that the levels of the toxic metals were insufficient to qualify as contaminated. By contrast, the soil Cd level was between 0 and 1, suggesting that these soils were uncontaminated to moderately contaminate. The *I*_*geo*_ values of soils were variable, with the maximum greater than 0 except for Cr, indicating that the toxic metals were present at different enrichments. According to abovementioned results, the contamination levels of these toxic metals decreased in the following sequence: Cd, Ni, Cr, Zn, Cu, and Pb.

**Table 4:** Geoaccumulation index.

Element	*I*_*geo*_				
	Minimum value	Maximum value	Average value	Number of points (>0)	Number of points (>1)
Cr	−1.09	−0.01	−0.47	0	0
Ni	−1.23	0.85	−0.26	19	0
Cu	−2.39	0.13	−0.69	2	0
Zn	−1.50	0.39	−0.55	3	0
Cd	−1.74	2.48	0.01	52	2
Pb	−1.38	0.57	−0.75	4	0

*I_*geo*_ ≤ 0 means the soil was practically uncontaminated; 0~1 means the soil was uncontaminated to moderately contaminated; 1~2 means the soil was moderately contaminated; 2~3 means the soil was moderately to heavily contaminated; 3~4 means the soil was heavily contaminated; 4~5 means the soil was heavily to extremely contaminated; >5 extremely contaminated.

The semivariograms of Cr, Ni, Cu, Zn, Cd and Pb matched the K-Bessel, Gaussian, stable, K-Bessel, exponential and stable models, respectively, as determined by semivariance analysis and the spatial distribution technique (Table 5). The prediction accuracy was acceptable, for all of the mean standardized (MS) values, which were close to 0, and for all of the root-mean-square standardized (RMSS) values, which were close to 1. All of the toxic metals met the condition of C_0_/(C + C_0_)(%) < 75%, which indicated strong or moderate horizontal spatial dependence. This pattern was especially evident for Cr, Ni and Pb, which had values < 25%.

**Table 5:** Theoretical models of semivariance and relevant parameters based on Kriging.

Item	Cr	Ni	Cu	Zn	Cd	Pb
Transformation	Logarithmic	/	/	/	Logarithmic	Logarithmic
Model	K-Bessel	Gaussian	Stable	K-Bessel	Exponential	Stable
C_0_	0	11.821	7.411	115.227	0.035	0.001
C + C_0_	0.043	53.260	28.03	233.263	0.099	0.056
C_0_/(C + C_0_)(%)	0	22.19	26.44	49.13	35.35	2.3
Range variation (m)	2665.1	768.7	1504.8	1872.3	415.9	955.6
Mean Standardized	0.0004	−0.0076	0.0029	0.0067	−0.0845	−0.0781
Root-Mean-Square Standardized	0.9416	1.0908	1.0630	1.0616	1.1310	1.2613

**Figure 1:**
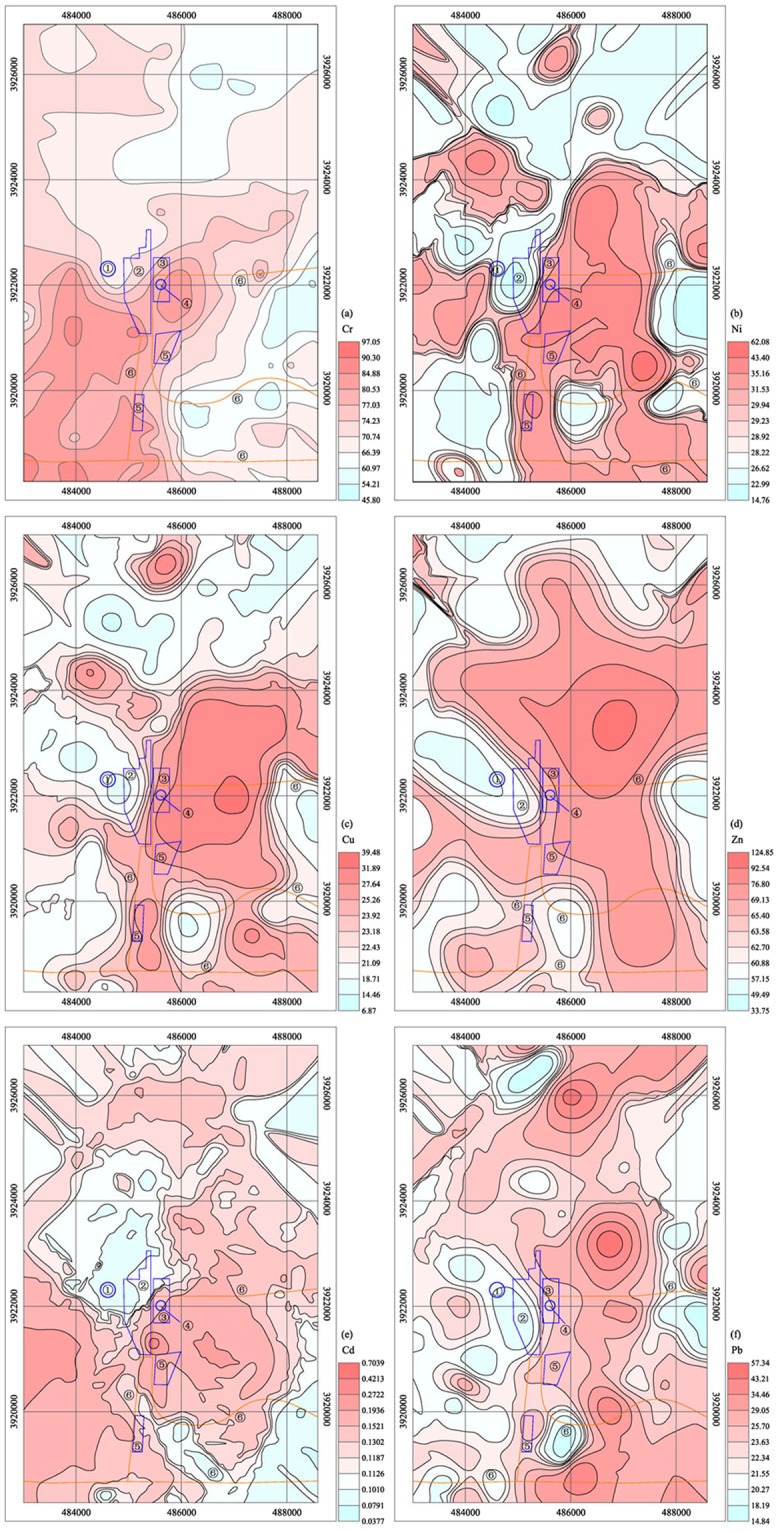
Spatial distribution maps of the toxic metals (Cr, Ni, Cu, Zn, Cd, and Pb) concentrations ①Coal gangue hill, ②Coal storage, ③Industrial square, ④Mineshaft, ⑤Transfer station, ⑥Main route for coal transportation.

The spatial variations of the toxic metal contents in soils are shown in Fig. 1. The average value and highest value of Cr were 72.16 and 97.05 mg/Kg, respectively, and the highest values were located in the middle of industrial square and the coal transport station. Overall, the spatial distribution trend for Cr in soils was low in the north and southwest of the study area but with obvious accumulation, continuous development and strong diffusion south of the gangue dump, south and east of the coal storage yard and the industrial square, and on both sides of the coal transfer station and coal transportation road. This result indicated that the high content of Cr mostly coincided with the coal mine and was mostly transported by surface water rather than atmospheric deposition, as surface water predominantly flowed from northwest to southeast, but wind was from the southeast. There have been a number of statements about Cr from coal mining^37,38^. Many studies have identified the enrichment of Cr in coal dust or fly ash in the coal mining area^39,40^. For example, it has been well documented that the Cr concentrations in leachates of fly ash in Sarigkiol basin^41^ accounted for more than 96% of the total Cr. Through this result, we can recognize that the soil Cr content in coal mine areas has a strong dispersal potential. The soil Cr concentrations were partly enhanced by coal mine activities, but not strongly.

The spatial variations of the Ni and Cu concentrations were consistent. The average value of Ni was 29.53 mg/Kg, and the average value of Cu was 23.07 mg/Kg. Both of their highest values were found near the coal transportation road. The Ni and Cu hotspots were south of the gangue dump, south and east of the coal storage yard and industrial square, and on both sides of the coal transfer station and coal transportation road. The spatial variations of these two elements were approximately the same as that of Cr. The only difference was the obvious accumulation on both sides of the coal transportation road in the northern area as the road passed through residential areas. This result indicates that the high contents of Ni and Cu mostly coincided with the coal mine and the discharging of domestic waste and that the transportation characteristics were similar to those of Cr. In addition to coal mining sources^42^, there have been a number studies that showing that Ni and Cu come from domestic waste^43,44^. Through this result, we can recognize that the soil Ni and Cu contents in coal mine areas have a strong dispersal potential. The soil Ni and Cu concentrations were enhanced by coal mine activities and domestic waste, but not strongly.

The average content of Cd was 0.14 mg/Kg, with the highest value of 0.70 mg/Kg located between the industrial square and coal transportation transit station. The Cd hotspots were highly concentrated around the industrial square. The soil Cd contents were high at the south of the gangue dump, south and east of the coal storage yard, eastern edge of the study area and on both sides of coal transportation road. On the contrary the Cd contents were low at the southwest edge of the study area. This result indicates that the high content of Cd mostly coincided with the coal mine and was partly affected by other human activities^45^. In contrast to Cr, Cd was transported by both surface water and atmospheric deposition^46^.

The average content of Zn was 66.30 mg/Kg, which was slightly higher than the soil background value of Shandong Province. The maximum value of the Zn content was 124.85 mg/Kg, located at the north side of the north coal transportation road. The Zn hotspots included areas south and east of the coal storage yard and industrial square and on both sides of the coal transfer station and coal transportation road. This result indicates that the high content of Zn mostly coincided with the discharge of domestic waste rather than coal mining activities. It was identified that high Zn loads were attributed to vehicular emissions and the wide use of Zn-coated building materials^47^. The Zn transportation characteristics were similar to those of Cd, with Zn transported by the combined actions of surface water and atmospheric deposition.

The mean value of Pb was 23.71 mg/Kg, and the highest value was 57.34 mg/Kg, which was located at the north side of the north coal road. The Pb hotspots included areas south and east of the industrial square and on both sides of the coal transfer station and coal road. The spatial variations of Pb were similar to those of Cu. This result indicates that the high content of Pb arises from several sources, including discharge of domestic waste, coal mine activities and road transportation^48^. The similar pollution sources of Zn and Pb, determined their similar spatial distribution characteristics. According to previous studies, the pollution of heavy metals such as Pb and Zn produced by vehicles is generally 150 m on both sides of the road^49,50^. The spatial distribution characteristics of Pb showed low diffusion levels, and the transportation characteristics were similar to t hose of Cd, with Pb primarily transported by the combined actions of surface water and atmospheric deposition.

### Micro-domain variation of toxic metals in the typical area of the coal transportation line

**Figure 2:**
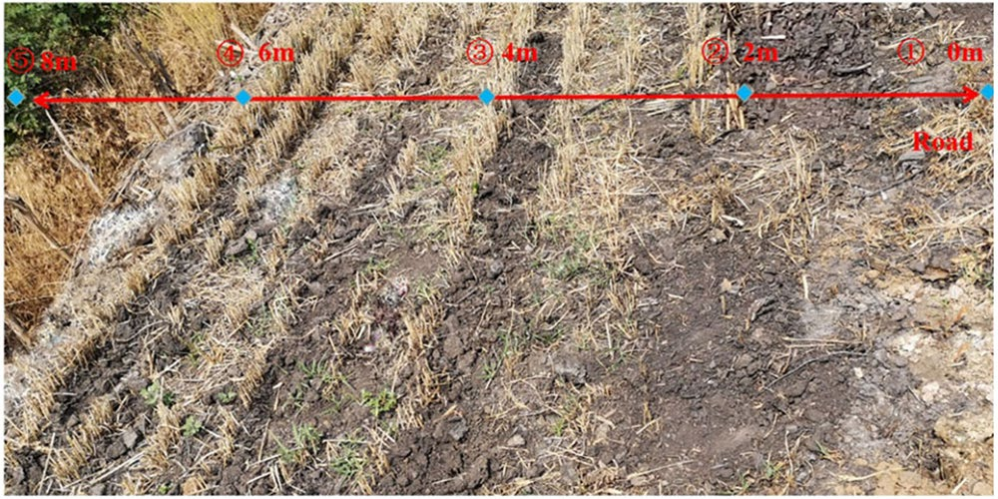
Changes in the soil toxic metal concentration at different distances from the roadside.

There are drains on both sides of the coal transportation road. A sprinkler sprays the road continuously to reduce road dust. Sewage from these drains is dumped directly onto roadside fields, which may increase the concentrations of toxic metals in soils. Figure 2 shows samples spaced 2 m away from the road, and Table 6 presents the values of toxic metals concentrations in the samples. The concentrations of soil toxic metals tend to gradually decrease from points ①-③ and ④-⑤ and sharply decrease from points ③-④. It is observed that the soil is darker and wheat straw is pale yellow at points ①-③. The wheat straw is sparse, with basically no weed growth, at point ①. The soil toxic metals concentrations are higher near the road than at other points far away from the road and are even higher than the concentrations in coal gangue, except for Zn. From points ② to ③, the wheat stalks are more consistent, increasingly more weeds appear, and the toxic metal concentrations slightly decrease. Wheat straw become bright yellow and dense at points ④ and ⑤. The soil toxic metals concentrations decrease slightly, but are still higher than those in coal, especially for Cu and Pb. It is observed that the soil toxic metal concentrations decrease from the roadside to farmland, where grows better. This result shows that there are obvious micro-domain variation characteristics in soil on both sides of the road due to the influence of coal transportation.

**Table 6:** Soil toxic metals concentrations in the typical area of coal transportation line.

Points	Toxic metals concentration (The average of three points) (mg/kg)					
	Cr	Ni	Cu	Zn	Cd	Pb
①	117.49	98.45	74.83	158.14	0.83	108.98
②	115.11	98.16	74.28	156.22	0.79	108.83
③	102.33	93.12	63.22	151.13	0.81	105.11
④	88.98	74.94	42.01	143.55	0.73	73.06
⑤	86.16	69.07	40.38	134.96	0.75	70.04

## Conclusions

The conclusions of this study are that the average concentrations of Cr, Ni, Zn, Cd and Pb are higher than their background contents in soils in Shandong Province, but the average concentrations of Cu and Pb are lower than their background levels. Additionally, the maximum concentrations of toxic metals are far above the background contents in soils in Shandong Province, particularly for Cd and Ni, which had maximum concentrations 8.3 and 2.7 times greater than their background values in Shandong Province, respectively. The soil concentrations of Ni and Cu have a positive and high correlation coefficient. Meanwhile, Ni-Zn and Cu-Zn also have positive correlation coefficients. Through geoaccumulation index analysis, the contamination levels of these toxic metals are shown to be low, though Cd and limited areas for other metals reached moderately contaminated levels. Generally, the contamination levels of toxic metals except for Cd can be regarded as practically uncontaminated. Through spatial distribution analysis, the six toxic metals were shown to have different levels of accumulation around the coal gangue dump, industrial square, and coal transfer station and transportation roads. This result indicates that the soil toxic metal contents are influenced by coal mine activities. In addition, Ni and Cu partially source from domestic waste, Cd partially comes from other human activities at the eastern edge of the study area, a majority of Zn sources come from the discharge of domestic waste, and Pb comes from domestic waste and road transportation sources. Through micro-domain variation analysis of toxic metals in a typical area of the coal transportation line, contamination by toxic metals is very serious on the sides of the coal transportation road. The abovementioned results are useful for the prevention and reduction of toxic metal contamination in soils and for providing a reference a for similar mining areas.

## Materials and Methods

### Soil sampling and analysis

The coal mine sampled is located in Zoucheng and Yanzhou, southwest of Shandong province, China. Its total area is 46.25 km^2^, and its designed annual capacity is 3.0 Mt. The mine has a warm temperate monsoon climate, where the average annual precipitation is 712.6 mm, and has a southerly prevailing wind. The mining site is low-lying, with a mean altitude of 40 m-46 m and gentle slope that decreases from northeast to southwest. The soil is rich regarding its ability to preserve water and nutrients and is dominated by meadow cinnamon soil, followed by lime concretion black soil. This site has experienced 31 years of mining activity that began in 1986. This site has complex topography, including plains, wetlands, rivers, and ponds because of coal mining subsidence and other human activities. According to satellite remote sensing images, a large amount of remediation has been performed. For example, drains around the coal gangue dump were added before 2010, and recycling activities had been gradually carried out until 2017. In addition, many reclamation projects of mining depressions have been performed.

**Figure 3:**
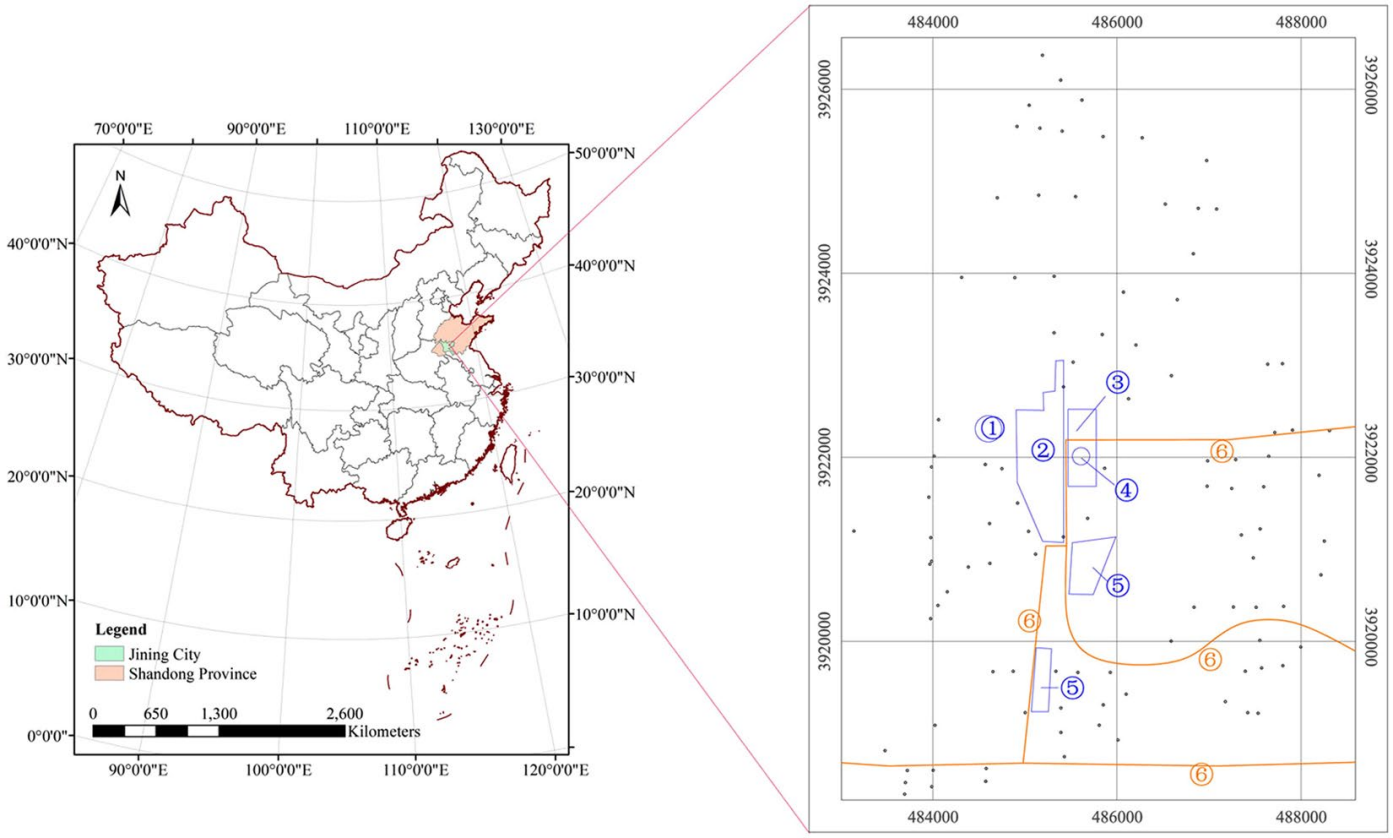
Location and sampling points distribution diagram ①Coal gangue hill, ②Coal storage, ③Industrial square, ④Mineshaft, ⑤Transfer station, ⑥Main route for coal transportation.

One hundred-three soil samples were collected from surface soil (0–20 cm depths) according to the distance (10 m, 50 m, 200 m, 500 m, 1000 m, 2000 m) for potential pollutant sources (gangue mountain, mine wellhead and coal transportation line) to assess the contamination potential and explore the spatial variation of the toxic metals. The sampling points were arranged in four directions from the gangue mountain and wellhead along both sides of the transport line and evenly distributed in the other areas^12,26^. The actual samples needed to be adjusted to avoid residential areas, reservoirs, rivers and villages and so on; two-thirds of which were arranged on the south side of the site, but the overall arrangement was also centered on the pollutants (Fig. 3). In addition, 15 soil samples on three lines were designed to be collected from surface soil (0–20 cm depths) according to the distance (0 m, 2 m, 4 m, 6 m, 8 m) from the coal transportation line to study the micro-domain variation at the typical area^51^.

Soil samples were collected following a five-point mixing sampling method and selected according to the quartering method. First, soil was dug out with a spade, and the portion that had not been exposed to the spade was placed in sample bags with disposable wooden spades. Second, all soil samples were dried naturally, and then, rocks and plant matter were removed. We broke clods using an agate ball mill to pass through a 200 mesh screen and mixed the soil completely and homogenously for later use. We measured the concentrations of Cr, Ni, Cu, Zn, Cd and Pb using a Nexion 300X inductively coupled plasma mass spectrometer (ICP-MS) (Perkin Elmer, USA) after a series of ‘Aqua regia’ digestions and acidification while maintaining a constant volume, which has been widely used to characterize trace elements concentrations in soil pollution studies^6,32^.

### Descriptive statistics

The standard deviation, coefficient of variation, mean value and media value were used to estimate the variability of the soil metal concentrations (Cr, Ni, Cu, Zn, Cd and Pb). The Kolmogorov-Smirnov (K-S) test together with asymptotic significance (double side), skewness, kurtosis values and a QQ-plot were used to determine the normality of the data. If ${{P}}_{k{-}s} > 0.05$, the hypothesis of the Kolmogorov-Smirnov (K-S) test that the data were normally distributed would be considered true. Correlation analysis was performed to identify the correlations between soil indicators and identify the interactions between soil indicators and distances to different pollutant sources. All data were entered into Microsoft Excel 2010 (Microsoft, Washington, USA), and statistical parameters were calculated using SPSS 16.0 (IBM SPSS Inc., Chicago, USA) for Windows.

### Contamination assessment and geostatistical analysis method

A pollution index, a geoaccumulation index *I*_*geo*_, was calculated to assess the contamination level of heavy metals in soil according to equation (1).

1 $${I}_{geo}={\mathrm{log}}_{2}(\frac{{C}_{i}}{1.5\times {B}_{i}})$$

where *C*_*i*_ is the concentration of the examined toxic element in soil and *B*_*i*_ is the background value of the element. In this study, we chose to use the soil background value in Shandong province as the background value.

Geostatistics were used to examine spatial autocorrelation and provide the input parameters for the spatial interpolation of kriging, which uses the semivariogram as a basic tool. The geostatistics approach consists of two parts, and more detailed information can be found in many monographs^19–21^. The first step is the calculation of the experimental semivariogram under the theory of intrinsic hypothesis using equation (2):

2 $$\hat{{\gamma }}({h}){=}\frac{{1}}{{2N}({h})}\sum _{i{=}1}^{N{(}h{)}}{[{Z}({{x}}_{{1}})-{Z}({{x}}_{{1}}+{h})]}^{{2}}$$

where $\hat{{\gamma }}({h})$ is the value of the semivariance for the lag interval *h*; *N*(*h*) is the number of pairs separated by a distance *h* that are used in the calculation of semivariance; *z*(*x*_1_) and ${Z}({{x}}_{{1}}+{h})$ are the values of the property at locations *x*_1_ and ${{x}}_{{1}}+{h}$, respectively.

There are different fitting models for the semivariogram, including Gaussian, stable, exponential, K-Bessel model and so on. The fitting model was chosen as the model that had the minimum sum of the squared deviations between the experimental and theoretical semivariograms. *C*_0_ and ${{C}}_{{0}}+{C}$ in the fitting models represent the nugget and the sill or total variance. The range variation is the range of spatial dependence. The nugget to sill ratio, ${{C}}_{{0}}{/}({C}+{{C}}_{{0}})({ \% })$, mainly represents the extent of spatial dependence, with values of <25%, 25–75% and >75% representing strong, moderate and weak spatial dependence, respectively.

The second step: Ordinary Kriging interpolation was applied to produce spatial distribution maps based on the spatial interpolation method. This method uses linear interpolation to estimate data from unknown samplings by the linear optimal unbiased estimation using equation (3). The *w*_*i*_ parameter can be determined using equation (4).

3 $${Z}^{\prime} ({{x}}_{0})=\sum _{i{=}1}^{n}{\omega }_{{i}}\cdot {Z}({{x}}_{{i}})$$

where the Kriging interpolation result ${Z}^{\prime} ({{x}}_{0})$ is the value of ${Z}({{x}}_{0})$ at *x*_0_ as a sum of weighted values of the known sampling points ${Z}({{x}}_{{i}})$.

4 $$\sum _{i{=}1}^{n}{\omega }_{{i}}\cdot {C}({{x}}_{{i}},{{y}}_{{j}})+\mu ={{C}}_{0}({{x}}_{{i}},{{y}}_{{j}}),{(}j=1{,}2\mathrm{......}{,}n{)},(\sum _{i{=}1}^{n}{\omega }_{{i}}=1)$$

## Data Availability

We confirm that the data supporting the findings of this study are available within the article and its supplementary materials.

## References

[1] Jia Z (2017). Comparison study on the estimation of the spatial distribution of regional soil metal(loid)s pollution based on kriging interpolation and BP neural network. Int. J. Environ. Res. Public Health.

[2] Ivanišević D, Malvić T (2016). Distribution of potentially toxic metals (As, Cu, Hg, Pb and Zn) in the topsoil of the Pannonian Basin System and associated parts of the surrounding orogens. J. Maps.

[3] Hasani S, Asghari O, Doulati Ardejani F, Yousefi S (2017). Spatial modelling of hazardous elements at waste dumps using geostatistical approach: a case study Sarcheshmeh copper mine, Iran. Environ. Earth Sci..

[4] Ministry of environmental protection of the People’s Republic of China, Ministry of land and resources of the People’s Republic of China (2014) Investigation communique on soil pollution in China. http://www.zhb.gov.cn/gkml/hbb/qt/201404/t20140417_270670.htm (2014).

[5] Panagos Panos, Van Liedekerke Marc, Yigini Yusuf, Montanarella Luca (2013). Contaminated Sites in Europe: Review of the Current Situation Based on Data Collected through a European Network. Journal of Environmental and Public Health.

[6] Li Z (2014). Science of the Total Environment A review of soil heavy metal pollution from mines in China: Pollution and health risk assessment. Sci. Total Environ..

[7] Achary MS (2017). Concentration of heavy metals in the food chain components of the nearshore coastal waters of Kalpakkam, southeast coast of India. Food Control.

[8] Dong J, Yu M, Bian Z (2012). The safety study of heavy metal pollution in wheat planted in reclaimed soil of mining areas in Xuzhou, China. Environmental Earth Sciences.

[9] Chen Y, Yuan L, Xu C (2017). Accumulation behavior of toxic elements in the soil and plant from Xinzhuangzi reclaimed mining areas, China. Environ. Earth Sci..

[10] Candeias C (2011). Assessment of soil contamination by potentially toxic elements in the aljustrel mining area in order to implement soil reclamation strategies. L. Degrad. Dev..

[11] Nicolau JM (2003). Trends in relief design and construction in opencast mining reclamation. L. Degrad. Dev..

[12] Ge H, Feng Y, Li Y, Yang WL, Gong N (2016). Heavy metal pollution diagnosis and ecological risk assessment of the surrounding soils of coal waste pile at Naluo Coal Mine, Liupanshui, Guizhou. International Journal of Mining Reclamation and Environment.

[13] Niu S, Gao L, Zhao J (2015). Distribution and risk assessment of heavy metals in the Xinzhuangzi reclamation soil from the Huainan coal mining area, China. Human and Ecological Risk Assessment.

[14] Xiuzhen T, Changyuan T, Pan W, Chipeng Z, Zhikang W (2017). Distribution and food exposure risk assessment of heavy metals inmature rice on the coal mining area, Guizhou TAO. Ecol. Environ. Sci..

[15] Jiali C, Xianhui Z, Zhewu T (2016). Contamination and health risk of heavy metals in vegetables from coal mining area in Huai’nan. J. Environ. Heal..

[16] Peiyou L, Yuehui S, Jiajian Z, Yong L (2015). Heavy metal component research in coal mine waste water of Bijie city. Energy Environ. Prot..

[17] Geng C, Jun L, Lihui Y, Zhaoyue K (2016). Pollution characteristics and health risk assessment of heavy metals in dust surrounding a coal-fired power plant. ACTA Sci. Nat. Univ. Sunyatseni.

[18] Chang L (2017). Spatial variability and contamination evaluation of arsenic in soils of Xijiang river basin. Environ. Sci..

[19] Domínguez MT (2016). River banks and channels as hotspots of soil pollution after large-scale remediation of a river basin. Geoderma.

[20] Wang J, Lu X, Feng Y, Yang R (2018). Integrating multi-fractal theory and geo-statistics method to characterize the spatial variability of particle size distribution of minesoils. Geoderma.

[21] Feng Y (2013). Soil heavy metal sources and pollution assessment in the coalfield of East Junggar Basin in XinJiang. China Environ. Sci..

[22] Dawei H, Herong G (2017). Sources analysis and content characteristics of soil heavy metal in Sunan mining area, China. Earth Environ..

[23] Shao L (2016). Pollution assessment and spatial distribution characteristics of heavy metals in soils of coal mining area in Longkou city. Environ. Sci..

[24] Qiao L, Shufen W, Youzhi C, Wei W, Chenglin H (2017). Ecological Risk Assessment and Source Analysis of Heavy-metal Pollution in Farmland Soils Surrounding the Coal Mine of East Junggar Basin, China. J. Agro-Environment Sci..

[25] Deyi R, Fenghua Z, Junying Z, Dewei X (1999). A preliminary study on genetic type of enrichment for hazardous minor and trace elements in coal. Earth Sci. Front..

[26] Xuren, W. Environmental geochemistry of heavy metal elements in coal mines of the western-south of Shandong province. (Wuhan University of Technology, 2012).

[27] Tao, G., Ping, C. & Xiuyi, T. Study on characteristic of mercury content and it’s transporting in washing-selecting of Huainan raw coal. In *International Mining Forum 2010:Mine Safety and Efficient Exploitation Facing Challenge of the 21st Century* 184 (2010).

[28] Mingyi R (2018). Spatial trends and pollution assessment for mercury in the surface soils of the Nansi Lake catchment, China. Environ. Sci. Pollut. Res..

[29] Shangguan Y, Wei Y, Wang L, Hou H (2016). Sources and distribution of trace elements in soils near coal-related industries. Arch. Environ. Contam. Toxicol..

[30] Wenjun W, Jin Z, Yingjian Z (2013). Investigation on heavy metals pollution in coal-mining subsided water of Jining City. Occup. Heal..

[31] Sheng Q, Liya T, Jian Z (2010). Speciation analysisi of trace elements in the waste piles and surrounding soil in Yanzhou mine field. China Coal.

[32] Hongxia L, Xiaoying W, Baoping H (2004). Soil pollution evaluation with heavy metals in baodian coal field of Yanzhou mineral industry group. Energy Environ. Prot..

[33] Liang J (2017). Spatial distribution and source identification of heavy metals in surface soils in a typical coal mine city, Lianyuan, China. Environ. Pollut..

[34] Pan H, Lu X, Lei K (2017). Science of the total environment a comprehensive analysis of heavy metals in urban road dust of Xi’an, China: Contamination, source apportionment and spatial distribution. Sci. Total Environ..

[35] Khadka D (2018). Soil fertility assessment and mapping of regional agricultural research station. Parwanipur, Bara, Nepal..

[36] Manta D, Angelone M, Bellanca A (2002). Heavy metals in urban soils: a case study from the city of Palermo (Sicily), Italy. Sci. Total Environ..

[37] Hussain, R., Luo, K., Chao, Z. & Xiaofeng, Z. Trace elements concentration and distributions in coal and coal mining wastes and their environmental and health impacts in Shanxi, China. *Environ. Sci. Pollut. Res. Int*. 1–19 (2018). 10.1007/s11356-018-2148-2 29732512

[38] Li C, Liang H, Wang S, Liu J (2018). Study of harmful trace elements and rare earth elements in the Permian tectonically deformed coals from Lugou Mine, North China Coal Basin, China. J. Geochemical Explor..

[39] Ishtiaq M (2018). Potential harmful elements in coal dust and human health risk assessment near the mining areas in Cherat, Pakistan. Environ. Sci. Pollut. Res..

[40] Espitia-Pérez L (2018). Geospatial analysis of residential proximity to open-pit coal mining areas in relation to micronuclei frequency, particulate matter concentration, and elemental enrichment factors. Chemosphere.

[41] Kazakis N (2018). Environmentally available hexavalent chromium in soils and sediments impacted by dispersed fly ash in Sarigkiol basin (Northern Greece). Environ. Pollut..

[42] Ma J (2018). Heavy metal removal from aqueous solutions by calcium silicate powder from waste coal fly-ash. J. Clean. Prod..

[43] Shao S (2015). Source identification and apportionment of trace elements in soils in the Yangtze river delta, China. Environ. Sci. Pollut. Res..

[44] Roubaud E (2018). Catalysis of the hydrogen evolution reaction by hydrogen carbonate to decrease the voltage of microbial electrolysis cell fed with domestic wastewater. Electrochim. Acta.

[45] Wang M (2018). Heavy metal contamination and ecological risk assessment of swine manure irrigated vegetable soils in Jiangxi province, China. Bull. Environ. Contam. Toxicol..

[46] Liu Y, Xing J, Wang S, Fu X, Zheng H (2018). Source-specific speciation profiles of PM2.5for heavy metals and their anthropogenic emissions in China. Environ. Pollut..

[47] Weerasundara L, Magana-Arachchi DN, Ziyath AM, Goonetilleke A, Vithanage M (2018). Health risk assessment of heavy metals in atmospheric deposition in a congested city environment in a developing country: Kandy City, Sri Lanka. J. Environ. Manage..

[48] Ghosh S, Rabha R, Chowdhury M, Padhy PK (2018). Source and chemical species characterization of PM10and human health risk assessment of semi-urban, urban and industrial areas of West Bengal, India. Chemosphere.

[49] Hong Z (2008). Distribution characteristics of soil heavy metal pollution at both sides of the roads along Shenyang-Dalian expressway. J. Meteorol. Environ..

[50] Jinfei, F. Characteristics and rules of heavy metal pollution on roadside soil and crop along highway. (Nanjing Agricultural University, 2010).

[51] Zhuoran W (2016). Spatial variation of soil water and salt and microscopic variation of soil salinity in summer in typical area of the Yellow River Delta in Kenli County. Acta Ecol. Sin..

[52] Wang JM, Liu WH, Yang RX, Zhang L, Ma JJ (2013). Assessment of the potential ecological risk of heavy metals in reclaimed soils at an opencast coal mine. Disaster Adv..

[53] Xin L (2018). Source apportionment of heavy metals in farmland soils around mining area based on UNMIX model. Environ. Sci..

[54] Li K (2017). Spatial analysis, source identification and risk assessment of heavy metals in a coal mining area in Henan, Central China. Int. Biodeterior. Biodegradation.

[55] Li H, Ji H (2017). Chemical speciation, vertical profile and human health risk assessment of heavy metals in soils from coal-mine brownfield, Beijing, China. J. Geochemical Explor..

[56] Alekseenko VA, Bech J, Alekseenko AV, Shvydkaya NV, Roca N (2018). Environmental impact of disposal of coal mining wastes on soils and plants in Rostov Oblast, Russia. J. Geochemical Explor..

[57] Das A (2018). Geochemical sources of metal contamination in a coal mining area in Chhattisgarh, India using lead isotopic ratios. Chemosphere.

